# Takotsubo Cardiomyopathy Triggered by the Death of Pets (Cats): Two Case Reports

**DOI:** 10.7759/cureus.10690

**Published:** 2020-09-28

**Authors:** Muhammad Hanif, Muhammad Adnan Haider, Qianlan Xi, Mukarram Jamat Ali, Muhammad Aslam Khan

**Affiliations:** 1 Internal Medicine, Khyber Medical College, Hayatabad Medical Complex, Peshawar, PAK; 2 Internal Medicine, Allama Iqbal Medical College/Jinnah Hospital, Lahore, PAK; 3 Internal Medicine, West China Hospital, Sichuan University, Chengdu, CHN; 4 Internal Medicine, King Edward Medical University, Lahore, PAK

**Keywords:** takotsubo cardiomyopathy, impella, cardiogenic shock

## Abstract

Takotsubo cardiomyopathy otherwise called stress cardiomyopathy, which results in debilitating of a segment of heart muscles, is a sort of non-ischemic cardiomyopathy, set off by stress. We describe two case reports of takotsubo cardiomyopathy triggered by the significant stressful event being the death of pet cats. The rare nature of the type of stressor and the manifestation is something to be considered by healthcare providers.

## Introduction

Takotsubo cardiomyopathy, also known as stress cardiomyopathy or apical ballooning syndrome, is considered a localized cardiac systolic dysfunction of the left ventricular apex and mid ventricle [1]. Takotsubo cardiomyopathy was first described by Satoh et al. [2] and Dote et al. [3] and was given the name "takotsubo" as this is the Japanese name for an octopus trap or pot, which shares a similar shape to that of the systolic apical ballooning. Takotsubo cardiomyopathy occurs in approximately 1% to 2% of patients who present with troponin-positive suspected acute coronary syndrome or suspected ST[A1] -elevation myocardial infarction [4-6]. Although emotional or physical strain is considered to play a key role, very little is known about the pathogenesis of the disease, and there have only been three reported cases of takotsubo cardiomyopathy triggered by the death of dogs [7-11]. Herein, we report two cases of takotsubo cardiomyopathy triggered by the death of pet cats.

## Case presentation

Case 1

A 56-year-old male patient with insignificant past medical history presented to the ED with acute onset of severe chest pain radiating to his shoulders, started while doing work. His chest pain was associated with diaphoresis, nausea, and mild dyspnea. He stated that he was under stress because of the loss of his cat a few days prior.

**Figure 1:**
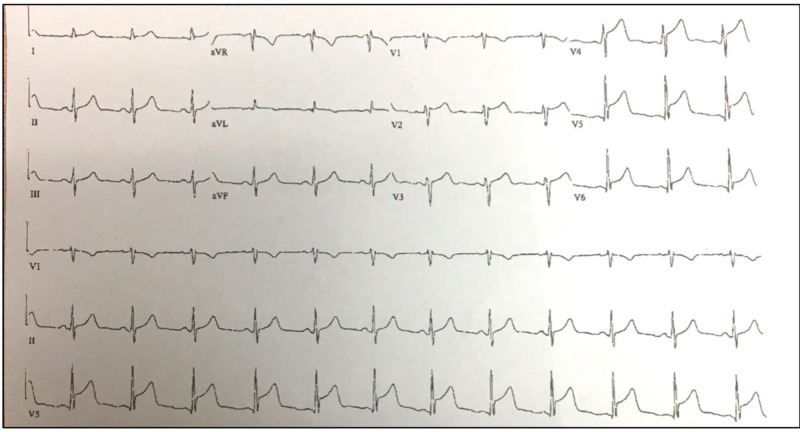
Thirty-three minutes after the onset of chest pain shows sinus anterolateral injury

**Figure 2:**
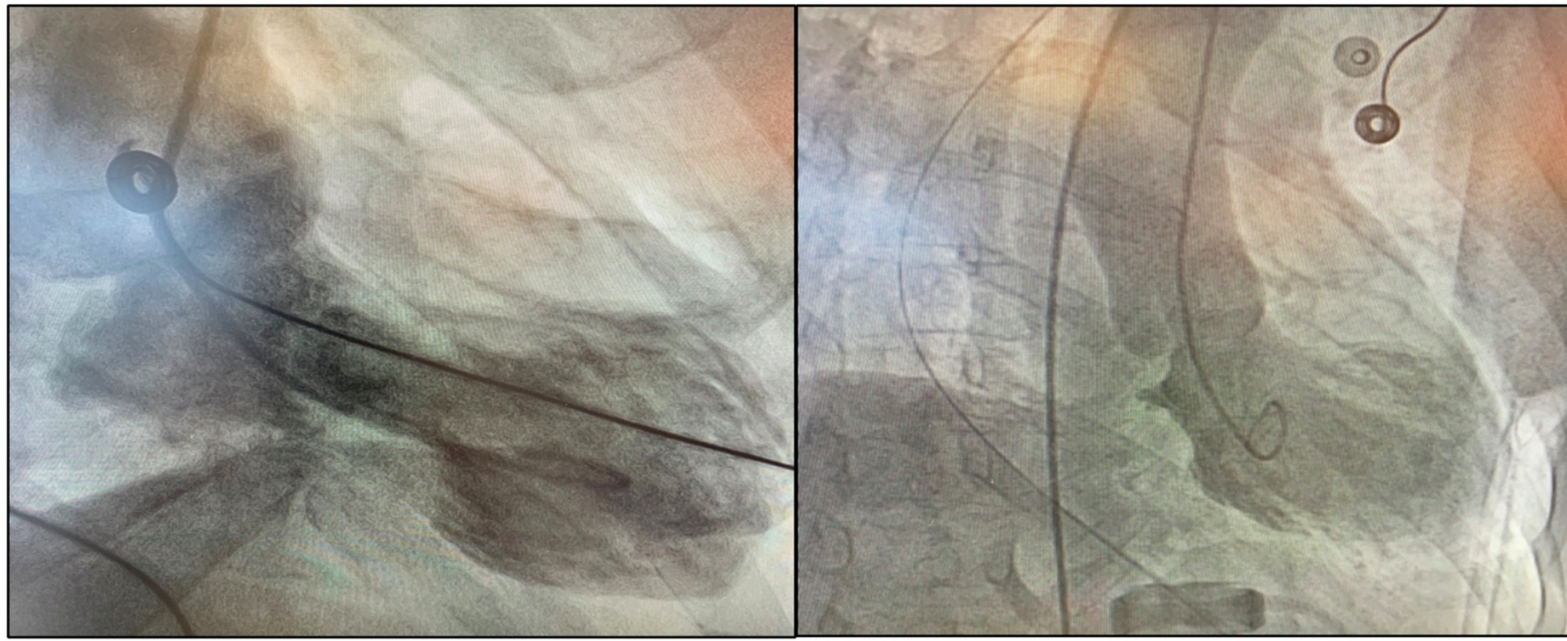
Left: cardiac catheterization showed hyperkinesia of base, hypokinesia of mid segment and apical segment was akinetic in case 1. Right: cardiac catheterization showed basal hyperkinesia and diffuse mid/apical akinesia in case 2

On examination, his blood pressure was 158/92 mmHg, his heart rate was 60 beats per minute, and his jugular venous pressure was elevated. The point of maximal impulse was situated outside the midclavicular line, S1 and S2 were within normal limits without any murmurs or gallops, and his lungs were clear. Serial ECG was performed on arrival, which showed ST-elevation in lead v4, v5, v6, and diagnosis of acute myocardial infarction was made based on EKG findings (Figure 1). Findings of cardiac catheterization were consistent with severely impaired left ventricular systolic function and no evidence of coronary artery occlusions (Figure 2, left). He was treated conservatively with aspirin, lisinopril, atorvastatin and bisoprolol as a case of takotsubo cardiomyopathy. Follow-up echocardiography performed after three months showed an improvement of ejection fraction from 30% to 60% with no regional wall motion abnormalities.

Case 2

A 54-year-old woman with known hypertension was brought to the ED unconscious. She had collapsed after being released from the hospital, and bystander cardiopulmonary resuscitation was administered. She stated that she was under stress because of the death of her cat two days prior.

On admission, the EKG showed ST-segment elevation with a high level of serum troponin. Echocardiogram with contrast showed severe systolic dysfunction with an estimated reduced left ventricular ejection fraction (LVEF) of 25%-29% and apical ballooning. Cardiac catheterization showed no coronary artery stenosis, along with a severely reduced LVEF, which was consistent with takotsubo cardiomyopathy (Figure 2, right).

The patient was admitted to the cardiac care unit and treated with intensive supportive treatments, including intubation with ventilation and right axillary Impella placed for severe cardiogenic shock. Endomyocardial biopsies showed myocyte hypertrophy, focal and mild interstitial fibrosis, no granuloma or inflammatory infiltrate, no amyloid deposition, no increased iron deposition, and positive immunofluorescent staining for IgG, IgA, IgM, C3, C4, C1q, and kappa and lambda light chains. On day 9 of hospitalization, her echocardiography showed improvement, with the LVEF rising to 50%-54%, and she was subsequently weaned off the Impella and mechanical ventilation.

After the application of the automatic implantable cardioverter defibrillators (AICDs), the patient was discharged on supportive therapy after 15 days of hospitalization. She was continually followed up as an outpatient. After three-month follow-up, echocardiography revealed normal wall motion with an increased ejection fraction of 60%-65%.

 

## Discussion

Transient takotsubo cardiomyopathy has been increasingly recognized since it was first described in Japan in 1990, and it thought to occur in 1.2% of patients with troponin-positive acute coronary syndrome (ACS) [4]. Although several factors have been suggested to play a role in the pathogenesis of takotsubo cardiomyopathy, the pathophysiologic mechanisms remain poorly understood. The study by Tsuchihashi et al. revealed that among all patients diagnosed with takotsubo cardiomyopathy, 20% had experienced some form of psychological stress, such as family accidents, deaths, or funerals [12]. The reason for the association between stressful events and takotsubo cardiomyopathy might be diffuse catecholamine-initiated microvascular spasm or dysfunction, resulting in myocardial stunning. The death of pets could also be a trigger, and has previously been reported with following the death of pet dogs [9-11]. The patients in our case report had emotional stress due to the loss of their pet cats. Thus, as the detection rate of takotsubo cardiomyopathy increases, the identification of possible triggers can help clinicians with early and accurate diagnosis.

Clinically, patients with takotsubo cardiomyopathy are similar to patients with ST-segment elevation myocardial infarction, but with the absence of obstructive lesions in coronary angiography [12]. The above-presented cases exhibited typical chest pain, raised ST segment, higher troponin, and ventricular wall motion anomalies, such as apical akinesia or dyskinesia, with maintained or increased contractile function of the basal LV segments from cardiac catheterization and echocardiography.

Although the base of the heart has an increased concentration of norepinephrine and a higher sympathetic nerve density compared to the apex, some studies suggest that the response to sympathetic stimulation from the apical myocardium can be greater; thus, the base of the heart may be more susceptible to abrupt catecholamine surges [13,14].

Takotsubo cardiomyopathy is normally a short-lived condition that can be treated with conservative therapy. Furthermore, decreasing physical or emotional trauma and supportive treatment can help with the quick resolution of symptoms. However, aggressive therapy is required in approximately 10% of patients who progress to acute complications such as shock and acute heart failure [15]. In patients who survive the acute episode, their systolic ventricular function normally recovers within one to four weeks [7,12,16].

In the study by Sharkey et al. [17], the mean LVEF increased from 29% at presentation to 63% at a mean of six days. In the study by Wittstein et al., the mean LVEF increased from a median of 20% at presentation to 60% at two to four weeks [7]. The Impella has been used successfully in patients with cardiogenic shock resulting from takotsubo cardiomyopathy [18,19].

## Conclusions

Takotsubo cardiomyopathy is caused by various everyday life stressors, including, in rare cases, the death of a pet. This association should be kept in mind when dealing with cases of takotsubo cardiomyopathy to assist with early diagnosis and proper management.
